# Is myopenia sufficient to diagnose sarcopenia?

**DOI:** 10.1093/oncolo/oyag197

**Published:** 2026-05-20

**Authors:** Erkan Topkan, Duriye Ozturk, Ugur Selek

**Affiliations:** Department of Radiation Oncology, Faculty of Medicine, Baskent University, Adana 01120, Turkey; Department of Radiation Oncology, Afyonkarahisar Health and Science University, Afyonkarahisar 03030, Turkey; Department of Radiation Oncology, School of Medicine, Koç University, Istanbul 34035, Turkey

Dear Editor,

We congratulate Chan and colleagues on their significant study, which aimed to identify the optimal cutoff for skeletal muscle index (SMI) in predicting severe chemotherapy-related toxicities and overall survival (OS) in 158 patients diagnosed with metastatic gastric cancer (GC).^1^ The SMI was assessed at the level of third lumbar vertebra (L3) through computed tomography (CT) scans conducted before the commencement of chemotherapy to evaluate the status of sarcopenia. Thirty patients (19.0%) had low SMI. The multivariable analysis demonstrated that a low SMI was an independent significant predictor of increased hematological (*P* = .001) and non-hematological (*P* = .003) toxicity, reduced overall survival (OS) (*P* = .011), a diminished response rate to chemotherapy (*P* = .045), and a lower likelihood of utilizing subsequent lines of treatment (*P* < .001). The results of this study are pivotal for advancing research on sarcopenia in metastatic GC patients. However, addressing a specific terminological issue, namely the synonymous use of myopenia (muscle mass loss) and sarcopenia and its potential implications, is essential, as it may facilitate a more explicit understanding of the findings presented and guide future research endeavors.

Myopenia is commonly utilized as a synonym for sarcopenia in related research, likely due to the relative ease of its assessment using radiological methods.^2^^,^^3^ However, it is crucial to acknowledge that sarcopenia constitutes a more complex and severe condition than myopenia.^4^^,^^5^ The European Working Group on Sarcopenia in Older People (EWGSOP) 2 report, published in 2019, defines sarcopenia as the coexistence of two conditions: dynapenia (muscle strength loss) and myopenia.^5^ According to this definition, the presence of dynapenia indicates that sarcopenia is likely, while the coexistence of myopenia confirms the diagnosis of sarcopenia. Thus, evaluating sarcopenia status solely by calculating the SMI using CT imaging data is inadequate for accurately defining sarcopenia, as it does not meet the established criteria.^4^^,^^5^

The inappropriate interchangeable usage of the terms myopenia and sarcopenia may lead to at least three unintended consequences. First, concerning sarcopenia research, while some pharmacological agents may effectively reduce myopenia and show positive results in clinical trials, they may not necessarily improve dynapenia. But, if myopenia and sarcopenia are considered synonymous, it could lead to misleading conclusions that the investigated drug is effective in treating or alleviating sarcopenia.^6^ Second, this misuse may also underrate dynapenia’s superior predictive ability over myopenia for adverse outcomes, including treatment intolerance related to sarcopenia, risk of hospitalization, and mortality.^7^ And third, since dynapenia does not consistently coexist with myopenia in all patients, this practice may artificially inflate the actual prevalence rates of sarcopenia among these individuals, compromise the validity of study outcomes, and complicate dependable comparisons between studies.^8–10^

In conclusion, despite assessment challenges, adhering to guideline definitions in sarcopenia research is imperative to facilitate consistent comparisons across diverse studies and enable a more accurate determination of the prevalence and impact of sarcopenia on patient outcomes. Finally, we believe appropriate use of myopenia will undoubtedly not reduce its established significance in oncological research and clinical settings but rather enhance the reliability and comparability of reported results.
